# Diffusion-weighted imaging of the female pelvis: how to avoid pitfalls

**DOI:** 10.1590/0100-3984.2018.51.2e3

**Published:** 2018

**Authors:** Alice Brandão

**Affiliations:** MD, PhD, Coordinator of the Imaging for Women Department of the Grupo Fleury, Radiologist at the Felippe Mattoso and Fonte Imagem Clinics, Rio de Janeiro, RJ, Brazil. E-mail: brandaosalomao@gmail.com

Diffusion-weighted imaging (DWI) is a noninvasive technique that reflects not only the histological structure of tissues but also the cellularity. DWI measures the random motion of free water molecules (Brownian motion). Unlike free-water diffusion, the movement of water molecules in biological tissues is restricted by interactions with cell membranes and macromolecules^(1)^.

Technological advances in magnetic resonance imaging (MRI) have been instrumental in broadening the use and applications of DWI. Currently, DWI is a sequence available on most MRI scanners; it is relatively fast and does not require exogenous contrast, making it a useful alternative for gadolinium contrast-enhanced sequences for the evaluation of patients at risk for progressive systemic fibrosis^(1,2)^.

On the basis of complementary information on microstructural properties of the tissue in the female pelvis, DWI facilitates the differentiation between benign and malignant pelvic tumors and has great sensitivity for the evaluation of peritoneal lesions as well as for the identification of tumor recurrence. Consequently, the technique has been routinely used in MRI scans of the pelvis. However, these advances have brought new challenges, because there are physiological and benign conditions of the female pelvis that can present restricted diffusion, despite not representing malignant tissue, as detailed and demonstrated by Duarte et al.^(3)^, in their article published in the previous issue of Radiologia Brasileira. The authors pointed out that 22% of female pelvic lesions exhibiting restricted diffusion are benign-either cystic lesions, such as abscesses, or solid lesions, such as cellular leiomyomas-with high signal intensity on DWI and low signal intensity on apparent diffusion coefficient (ADC) mapping.

Duarte et al.^(3)^ also describe physiological pelvic conditions, such as those observed in the endometrium and myometrium, which can show variable ADCs throughout the menstrual cycle and different ADCs for women using oral contraceptives. Normal ovaries can also exhibit high signal intensity on DWI, especially during the luteal phase.

How can we avoid the pitfalls related to physiological conditions and benign tissues? Certain precautions should be taken in order to avoid misinterpretation of DWI sequences, making it essential to correlate the DWI findings with those of T1-weighted sequences, with and without fat suppression, and T2-weighted sequences. Duarte et al.^(3)^ highlight several examples of such pitfalls. The authors of a study characterizing lesions in the uterus highlighted the behavior of hyalinized uterine leiomyoma, in which collagen deposition results in the "T2 blackout effect", decreasing signal intensity in DWI sequences and in ADC mapping^(4)^. However, highly cellular leiomyomas, in which there is little collagen deposition, show high signal intensity on DWI and low signal intensity on ADC mapping, similar to a uterine STUMP (smooth muscle tumor of uncertain malignant potential) and leiomyosarcoma. In this scenario, neither morphological sequences nor DWI are able to exclude malignancy, although they can indicate which nodule has the highest risk of atypia. Some authors have defined malignant tumors as those with a hyperintense signal in DWI and T2-weighted sequences and a hypointense signal on ADC mapping^(5-7)^, with an ADC below 0.97, an accuracy of 94%, a specificity of 94%, a sensitivity of 100%, and a negative predictive value of 100%, albeit with a positive predictive value of 64%.

Among ovarian lesions, fibromas, cystadenofibromas, and Brenner tumors present a solid component with a predominance of collagen, showing a hypointense signal in T2-weighted sequences and low signal intensity on DWI, findings that are considered strongly indicative of benignity, with a positive predictive value of 100%^(8)^.

Duarte et al.^(3)^ further note that benign ovarian lesions can mimic malignancy on DWI because of other factors, such as intracellular or extracellular edema (due to torsion with infarction), bleeding (due to endometrioma or a hemorrhagic corpus luteum cyst), and increased viscosity (due to teratoma or abscess). Therefore, in this context, the clinical implications of DWI depend on the tissue under investigation and the interpretation of the images must begin with the conventional sequences^(9-11)^.

As emphasized by Duarte et al.^(3)^, adding functional DWI to the conventional imaging study allows us to evaluate the microvascular characteristics and cellularity of lesions in the female pelvis, improving the differentiation between benign and malignant lesions, thus increasing the specificity of MRI. However, the DWI findings must be interpreted with caution and should always be correlated with the findings of conventional anatomical sequences. The interpretation of DWI findings in isolation can lead to false-positive results.
